# Liangxue Qushi Zhiyang Decoction Inhibits Atopic Dermatitis in Mice via Fc*γ*R-Mediated Phagocytosis

**DOI:** 10.1155/mi/7068964

**Published:** 2025-04-27

**Authors:** Lili Zhang, Linxian Li, Zhanxue Sun

**Affiliations:** Beijing University of Chinese Medicine Third Affiliated Hospital, Beijing, China

**Keywords:** Atopic dermatitis, Fc gamma R−mediated phagocytosis, Proteomics, Traditional Chinese medicine

## Abstract

**Background:** Liangxue Qushi Zhiyang Decoction (LQZ) is a traditional formula known for its efficacy in treating Atopic Dermatitis (AD). However, the specific mechanisms through which LQZ alleviates AD symptoms remain largely unknown. The objective of this study is to investigate the protective effects of LQZ on AD and to uncover its potential mechanisms of action.

**Methods:** An AD model was established in mice using 2,4-dinitrochlorobenzene (DNCB). Mice were then orally administered LQZ or prednisolone (PDN). Throughout the treatment period, dermatitis scores and scratching frequencies of the mice were regularly monitored. Histopathological analyses were conducted using hematoxylin and eosin (H&E) staining and toluidine blue (TB) staining. Serum levels of inflammatory cytokines were measured using enzyme-linked immunosorbent assay (ELISA). Further, tandem mass tag (TMT) labeling quantitative proteomics was employed to identify differentially expressed proteins (DEPs). Enrichment analysis was conducted to pinpoint potential targets and pathways involved in LQZ's therapeutic action. Finally, validation experiments were performed to further explore the specific pathways and core targets of LQZ in AD treatment..

**Results:** LQZ treatment notably mitigated the skin barrier damage and inflammatory response induced by DNCB in AD mice, and reduced the serum levels of IgE, IL-4, and IL-1*β*. Proteomic analysis identified 248 proteins with differential expression, implicating multiple pathways in LQZ' therapeutic action. Among these, the Fc gamma R(Fc*γ*R)−mediated phagocytosis pathway emerged as a crucial factor in AD's inflammatory and immune responses. Key proteins associated with this pathway, including Fc-gamma RIII (Fcgr3), V-yes-1 Yamaguchi sarcoma viral related oncogene homolog (Lyn), Tyrosine-protein kinase (Syk), Phosphoinositide phospholipase C-gamma-2 (Plcg2), Neutrophil cytosol factor 1 (Ncf1), Ras-related C3 botulinum toxin substrate 2 (Rac2) and Actin-related protein 2/3 complex subunit 3 (Arpc3), exhibited significantly reduced expression levels following LQZ treatment.

**Conclusion:** LQZ is effective in treating AD by alleviating skin barrier damage and inflammatory reactions. Its anti-AD properties of LQZ may be attributed to the inhibition of the Fc*γ*R-mediated phagocytic pathway.

## 1. Introduction

Atopic dermatitis (AD), a prevalent inflammatory dermatological disorder, is chiefly marked by severe pruritus and recurring eczema-like eruptions [1]. It is notably widespread in affluent nations, affecting up to 20% of children and 10% of adults [2]. Although its prevalence has plateaued in numerous affluent countries, its incidence is escalating in lower and middle-income nations [2]. The intricate etiology of AD involves impaired epidermal barrier function, altered cutaneous microbiota, and immune imbalances, predominantly driven by type 2 immune reactions [3]. Specifically, a compromised skin barrier, often due to keratin defects, allows for increased inflammation and T-cell infiltration. Colonization or infection by *Staphylococcus aureus* not only damages skin barrier but also triggers inflammatory responses. Local T helper 2 cells (Th2) immune responses further weaken the barrier, intensify itching, and lead to skin microbiome imbalances, favoring staphylococcal growth. Stress-induced alarmins like IL-33 and TSLP activate skin epithelial dendritic cells and type 2 responses. Subsequently, Th2 cells secrete IL-4 and IL-13, which promote B-cell IgE class switching and specific IgE synthesis through the STAT pathway [4].

In the management of AD, the principal objective is to diminish symptoms and sustain long-term control of the disorder. This includes avoiding triggers, restoring the skin barrier, and employing anti-inflammatory therapies [5]. Topical therapies, including moisturizers and corticosteroids, are crucial. Corticosteroids are relatively safe when used properly but can cause side effects if misused [6]. Systemic therapy is advised when topical treatments fail. For moderate-to-severe AD, corticosteroids, and immunosuppressants have traditionally been employed in systemic therapy [7]. However, prolonged steroid use can result in various side effects, including hypertension, obesity, diabetes, and hirsutism. Furthermore, intermittent steroid use can also lead to relapses and potentially worsen symptoms. Immunosuppressants generally require a longer duration to become effective and are associated with a higher occurrence of adverse events. Dupilumab, targeting IL-4R*α*, offers a new biologic option for moderate-to-severe cases, and oral JAK inhibitors have shown promise in clinical outcomes due to their multi-pathway immune modulation [8, 9]. Nevertheless, uncertainties remain regarding the long-term management and therapeutic approaches for AD, particularly concerning the sustained safety and efficacy of systemic and novel treatments [10].

Liangxue Qushi Zhiyang Decoction (LQZ) is a classical Traditional Chinese Medicine (TCM) formula traditionally used to treat eczema and AD, particularly for the AD type associated with blood-heat and dampness accumulation syndrome [11]. This syndrome is characterized by symptoms such as erythema, edema, exudation, and intense itching, which are commonly observed in acute stages of AD. According to TCM theory, blood-heat and dampness are considered common pathogenic factors that contribute to the development of AD [12]. The accumulation of these factors in the skin is believed to be the primary driver of AD pathogenesis. As such, LQZ is thought to exert therapeutic effects by cooling the blood, expelling dampness, and relieving itching, which are crucial for alleviating the associated symptoms. LQZ consists of 11 botanical ingredients, including *Bubali cornu* (*Bubalus bubalis Linnaeus*), *Rehmannia glutinosa* (*Gaertn*.) *DC*. (*Orobanchaceae*), *Sophora flavescens Aiton* (*Fabaceae*), *Paeonia lactiflora Pall*. (*Paeoniaceae*), *Coix lacryma-jobi var. ma-yuen* (*Rom.Caill*.) *Stapf* (*Poaceae*), *Imperata cylindrica* (*L*.) *Raeusch*. (*Poaceae*), *Lycopus lucidus Turcz. ex Benth*. (*Lamiaceae*), *Bassia scoparia* (*L*.) *A.J.Scott* (*Amaranthaceae*), *Paeonia × suffruticosa Andrews* (*Paeoniaceae*), *Poria* (*Poria co*cos (*Schw*.) *Wolf*), and *Glycyrrhiza glabra L*. (*Fabaceae*). These plants have been validated through World Flora Online (https://www.worldfloraonline.org).

Early experimental investigations utilizing UPLC-ESI-MS/MS and network pharmacology have identified 49 components absorbed into the serum from the LQZ aqueous extract [13]. Predominantly, these compounds are categorized into four groups: amino acids and their derivatives (e.g., L-arginine and L-phenylalanine), alkaloids (e.g., matrine and sophocarpine), lipids (e.g., *α*-hydroxylinoleic acid), and phenolic compounds (e.g., caffeic acid) [13]. Clinical studies have demonstrated that LQZ effectively alleviates AD symptoms, including erythema, edema, and pruritus. In a clinical trial, patients treated with LQZ showed a significantly higher total effective rate (87.50%) than those treated with cetirizine (46.88%) (*p* < 0.01) [11]. Another study showed that LQZ combined with boric acid lotion significantly outperformed loratadine plus boric acid lotion in reducing serum IL-13 levels (*p* < 0.05) and improving Dermatology Life Quality Index scores [14]. Initial experimental findings have demonstrated that LQZ exerts multiple beneficial effects on skin lesions in an AD mouse model. These include reducing erythema, alleviating edema, promoting epidermal repair, and preventing lichenification [13]. Moreover, it has been observed to modulate key inflammatory factors, such as elevating serum IL-2 levels, counteracting IL-4, and suppressing leukotriene B4 production [15]. These findings indicate that LQZ may play a pivotal regulatory role in AD therapy.

However, due to its holistic, multicomponent, and multi-target nature, the precise mechanisms by which LQZ exerts its anti-AD effects through various signaling pathways remain largely unexplored. This study represents the first attempt to elucidate the biological mechanisms underlying LQZ's efficacy from the perspectives of proteomics and molecular biology.

Quantitative proteomics is extensively utilized for identifying differentially expressed proteins (DEPs) in various disease pathologies. Its vital role extends beyond animal models to include clinical samples. With its superior screening capabilities, this approach has gained popularity in exploring the regulatory mechanisms of numerous traditional Chinese botanical metabolites. Thus, this study aims to leverage tandem mass tag (TMT)-based quantitative proteomics to uncover the therapeutic effects of LQZ, identify key signaling pathways, and pinpoint specific target proteins modulated by LQZ.

## 2. Materials and Methods

### 2.1. Preparation of LQZ

The freeze-dried LQZ powder used in this study was prepared under standardized protocols, identical to those described in our previous work [13]. Both the current experiment and prior studies utilized the same large-scale batch of LQZ, ensuring uniformity across experiments. The botanical drugs for this study were sourced from Dongfang Hospital, which is affiliated with Beijing University of Chinese Medicine. To prepare LQZ, the herbs were soaked in purified water (1:10 w/v) for 1 h, and then boiled twice at 100°C for 30 min each. After boiling, the decoction was filtered to remove residues, resulting in the extraction of the botanical essence. This extract was then concentrated using a rotary evaporator at 45°C and stored at −20°C for 24 h. The final preparation involved freeze-drying and sterilizing the sample with a lyophilizer, after which the sample was subpackaged and stored at −80°C. For experimental purposes, the LQZ was reconstituted with distilled water to achieve the desired concentrations of LQZ-L (7.6 g/kg) and LQZ-H (15.2 g/kg), and stored at −4°C.

### 2.2. Animal and Grouping

We utilized 30 female BALB/c mice, aged 8 weeks and weighing between 20 ± 2 g, which were acquired from Beijing Sibeifu Biotechnology Co., Ltd. These mice were randomly assigned into five groups, with each group consisting of six mice. The designated groups were: Control (CON), AD model (DNCB), AD model treated with 8 mg/kg prednisolone (PDN), AD model treated with 7.6 g/kg LQZ (LQZ-L), and AD model treated with 15.2 g/kg LQZ (LQZ-H). All the mice were housed in specific pathogen-free (SPF) conditions, with the ambient temperature maintained at 22 ± 2°C, and were provided with standard purified water and food. The experimental study received approval from the Ethics Committee of Beijing University of Chinese Medicine, with the approval date and number being 20230420 and BUCM-4-2023020705-1092, respectively.

### 2.3. DNCB-Induced AD Mice Construction and Intervention

**Figure 1:**
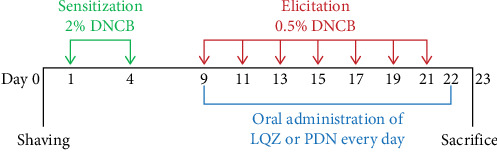
Illustration of in vivo anti-inflammatory study of LQZ in the DNCB-induced atopic dermatitis mouse model (*n* = 6). After 2% DNCB sensitization and 0.5% DNCB elicitation or acetone and olive oil treatment, mice were orally administrated with LQZ-L (7.6 g/kg), LQZ-H (15.2 g/kg), PDN (Prednisolone, 8 mg/kg, positive drug), or 0.9% saline once a day for 14 days.

We established a mouse model of AD using 2,4-dinitrochlorobenzene (DNCB). Initially, on day 0, we shaved a specific area (2 cm × 3 cm) on the back of each mouse. For the sensitization phase, on days 1 and 4, we applied 150 μL of 2% DNCB, dissolved in a 3:1 mixture of acetone and olive oil, to the shaved area on the mice's backs. In the elicitation phase, spanning from day 9 to day 22, we administered 150 μL of 0.5% DNCB to the same dorsal skin area every other day, along with 20 μL of DNCB to both right ears, repeating this application seven times. The CON group received the acetone and olive oil mixture without DNCB for both sensitization and elicitation phases. Starting from day 9, the LQZ-L and LQZ-H groups were orally given 7.6 and 15.2 g/kg of LQZ, respectively, while the PDN group was administered 8 mg/kg of PDN, 0.2 mL/20g, continuously for 14 days. The control and model groups were orally given an equivalent volume of saline for the same duration.  Figure 1 displays the flowchart of our experimental procedures.

### 2.4. Calculate Dermatitis Scores and Scratching Frequency

To evaluate the severity of the skin lesions, we used the dermatitis scores. From day 10 to day 22 of the elicitation phase, we captured photographs of the dorsal skin lesions on the mice every other day using a high-definition digital camera, enabling us to track dynamic changes. The scoring criteria were based on erythema, edema, epidermal peeling, and scales on the mice's backs, categorized into four grades: 0 (none), 1 (mild), 2 (moderate), and 3 (severe). Furthermore, on day 22, we recorded a 10-min high-definition video to observe the mice's scratching behavior. We counted the number of times each mouse scratched within this 10-min duration. These dermatitis scores and behavioral observations were instrumental in assessing disease severity and symptom manifestation in our AD mouse model.

### 2.5. Animal Sample Collection

On the 23rd of the experiment, we anesthetized the mice via intraperitoneal injection with 1% sodium pentobarbital at a dosage of 50 mg/kg. Blood samples were then collected through eyeball enucleation. These samples were left at room temperature for 2 h and then centrifuged at 3000 rpm for 15 min. The resulting supernatant was transferred into new 2 mL eppendorf tubes and stored at −80°C for future Enzyme-Linked Immunosorbent Assay (ELISA) testing. Following this, the mice were euthanized using rapid cervical dislocation. We collected skin samples from the dorsal skin and the right ear for further processing. Part of these samples was fixed in 4% paraformaldehyde for histopathological studies, while the rest was stored at −80°C for subsequent ELISA and TMT-based quantitative proteomics analysis.

### 2.6. Skin Histopathology Observation and Evaluation

The skin and ear tissue samples collected from the mice were fixed in 4% paraformaldehyde and then embedded them in paraffin to prepare sections 4 μm thick. These sections underwent histopathological staining with hematoxylin and eosin (H&E) and Toluidine Blue (TB). Each section was meticulously examined under a microscope at a magnification of 200×. H&E staining enabled the evaluation of the epidermal thickness in the mouse back and ear skin, and this was followed by statistical analysis. TB staining was employed specifically to identify mast cell infiltration in the dermal layers of the back and ear, allowing for the quantification of mast cell numbers.

### 2.7. ELISA

To analyze the levels of IgE, IL-4, and IL-1*β* in the mouse serum samples, we utilized the double antibody sandwich method, strictly following the protocols provided in the ELISA kit manual (Jiangsu Meibiao Biotechnology Co., Ltd., Yancheng, Jiangsu, China). The back skin tissue samples of the mice were weighed and then carefully minced using ophthalmic scissors. These minced tissues were placed in 0.01 mol/L PBS. We used a tissue homogenizer under ice bath conditions to homogenize the tissues at a 9 mL/g ratio. The homogenized mixture was then centrifuged at 3000 rpm for 10 min at 4°C, after which the supernatant was collected. Next, the total protein content in the tissue samples was quantitatively measured using a BCA protein assay kit (Jiangsu Meibiao Biotechnology Co., Ltd., Yancheng, Jiangsu, China). Subsequently, to assess the expression of specific proteins involved in Fc gamma R (Fc*γ*R)−mediated phagocytosis, including Fc-gamma RIII (Fcgr3), V-yes-1 Yamaguchi sarcoma viral related oncogene homolog (Lyn), Tyrosine-protein kinase (Syk), Phosphoinositide phospholipase C-gamma-2 (Plcg2), Neutrophil cytosol factor 1 (Ncf1), Ras-related C3 botulinum toxin substrate 2 (Rac), and Actin-related protein 2/3 complex subunit 3 (Arpc3), we employed an ELISA kit (Jiangsu Meibiao Biotechnology Co., Ltd., Yancheng, Jiangsu, China).

### 2.8. TMT-Based Quantitative Proteomics Analysis

#### 2.8.1. Protein Preprocessing

The proteomics experimental procedures were conducted based on previously published protocols, with minor modifications [16]. Protein samples were extracted from three pieces of back skin tissue from each group (CON, AD, PDN, and LQZ-H) using the Total Protein Extraction Kit for Mammalian Tissues (Beijing Bangfei Bioscience Co., Ltd., AP0601-50). The concentration of protein supernatants was determined following the BCA kit instructions. Based on this, the required protein amount for loading was calculated. For each group, 20 μg of protein sample was loaded and subjected to SDS-PAGE at 120 V for about 60–90 min. Subsequently, Coomassie Brilliant Blue staining was performed. The protein quality was then assessed using digital gel image analysis.

#### 2.8.2. Protein Digestion

An appropriate volume of protein solution was combined with 5 μL of 1M DTT and incubated at 37 °C for 1 h. Then, 20 μL of 1M IAA solution was added and the mixture was left to react in the dark at room temperature for 1 h. Detergents, DTT, and other low molecular weight components were removed via repeated ultrafiltration (10kD) using UA buffer (8M urea). The filter was washed twice with 100 μL UA buffer, followed by three washes with 100 μL of 0.5 M TEAB buffer. Each sample was digested at 37°C with a trypsin-to-protein concentration ratio of 1:50 for 12–16 h, resulting in the digested peptide solution. The peptides were then desalted using a C18 Cartridge.

#### 2.8.3. TMT Labeling

Following the protocol provided by Thermo Fisher Scientific, the peptide mixture from each sample was labeled with TMT reagents.

#### 2.8.4. Reverse Phase (RP) Chromatography for Peptide Fractionation

The TMT-labeled peptide mixtures underwent fractionation through RP chromatography using an HPLC system. Initially, the peptide mixtures were diluted with buffer A (2% acetonitrile, pH 10.0) and loaded onto an xBridge Peptide BEH 130 C18 column. Elution of peptides was then performed with buffer B (98% acetonitrile, pH 10.0) at a flow rate of 0.7 mL/min. Post-elution, the fractions were dried using a vacuum centrifuge at 45 °C.

#### 2.8.5. Liquid Chromatography-Tandem Mass Spectrometry (LC-MS) Analysis

Each fraction was analyzed via nanoLC-MS/MS. The peptide mixtures were loaded onto a C18-reverse phase analytical column (Thermo Fisher Scientific, San Jose, CA, USA). Isocratic elution was carried out using buffer A (0.1% formic acid in water), followed by separation at a flow rate of 600 nL/min using a linear gradient of buffer B (0.08% formic acid, 80% acetonitrile in water). The specific parameters of the linear gradient are shown in Table 1. Each sample underwent separation using μHPLC and was subsequently analyzed with an Orbitrap Fusion mass spectrometer (Thermo Scientific) over 78 min. Operating in positive ion mode, the mass spectrometer utilized a data-dependent top 10 method, dynamically selecting the most abundant precursor ions from the range of 350–1400 m/z for MS data acquisition. Survey scans were conducted at a resolution of 120,000, with an Automatic Gain Control (AGC) target set at 5e5 and a maximum injection time (IT) of 100 milliseconds. MS2 scans were collected at a resolution of 50,000 at 100 m/z, with a maximum IT of 86 milliseconds and an isolation window of 1.6 m/z. The normalized collision energy was set at 32 eV.

#### 2.8.6. Proteomics Analysis

The MS/MS raw files were processed and analyzed using Proteome Discoverer 2.5 software (Thermo Fisher Scientific, San Jose, CA, USA), with a search conducted against the human protein database from UniProt. Protein identification parameters included a peptide mass tolerance of ±10 ppm, an MS/MS tolerance of 0.02 Da, trypsin as the enzyme with up to 2 missed cleavages, and several modifications: fixed modifications of TMTpro (*N*-term) and TMTpro (K), carbamidomethyl (C), and variable modifications of oxidation (M) and acetylation (protein N-term). The false discovery rate (FDR) for peptides and proteins was set at 0.01. Proteins were considered differentially expressed if they exhibited a relative quantitative *p* − value  < 0.05 and a fold change ≥1.5 in at least two replicates.

Initially, all protein sequences were aligned to the NCBI database using ncbi-blast-2.2.28+-win32.exe, retaining only the top 10 sequences with an *E*-value ≤ 1e^–3^. The Gene Ontology (GO) term (database version: go_201504.obo) corresponding to the sequence with the highest Bit-Score was selected via Blast2GO. Protein annotation was completed through the Blast2GO Command Line. After preliminary annotation, InterProScan was utilized to search the EBI database for motifs, enhancing the functional annotation of the proteins by adding motif information. Further annotation refinement and linkage between GO terms were achieved using ANNEX. To enrich GO terms, Fisher's Exact Test was employed by comparing the number of DEPs against total proteins associated with specific GO terms. Pathway analysis was performed using the KEGG database, where Fisher's Exact Test identified significantly enriched pathways by comparing DEPs against total proteins linked to these pathways. For GO term enrichment analysis of key targets, Cytoscape's ClueGO and CluePedia plug-ins were used. The ClueGO enrichment analysis was configured with the following parameters: “Show only Pathway with *p*V ≤ 0.05, min level = 6, and max level = 6 in GO Tree Interval, min #genes = 3, and %genes = 4 in GO Term/Pathway Selection (#%Genes).“

### 2.9. Statistical Analysis

For the statistical analysis of our data, we utilized GraphPad Prism 10 to conduct one-way ANOVA. All data were presented as mean ± standard deviation (SD), and set the threshold for statistical significance at a *p*-value of ≤ 0.05. Additionally, we employed R (version 4.3.2) for further data analysis and visualization.

## 3. Results

### 3.1. LQZ Improved DNCB-Induced AD

**Figure 2:**
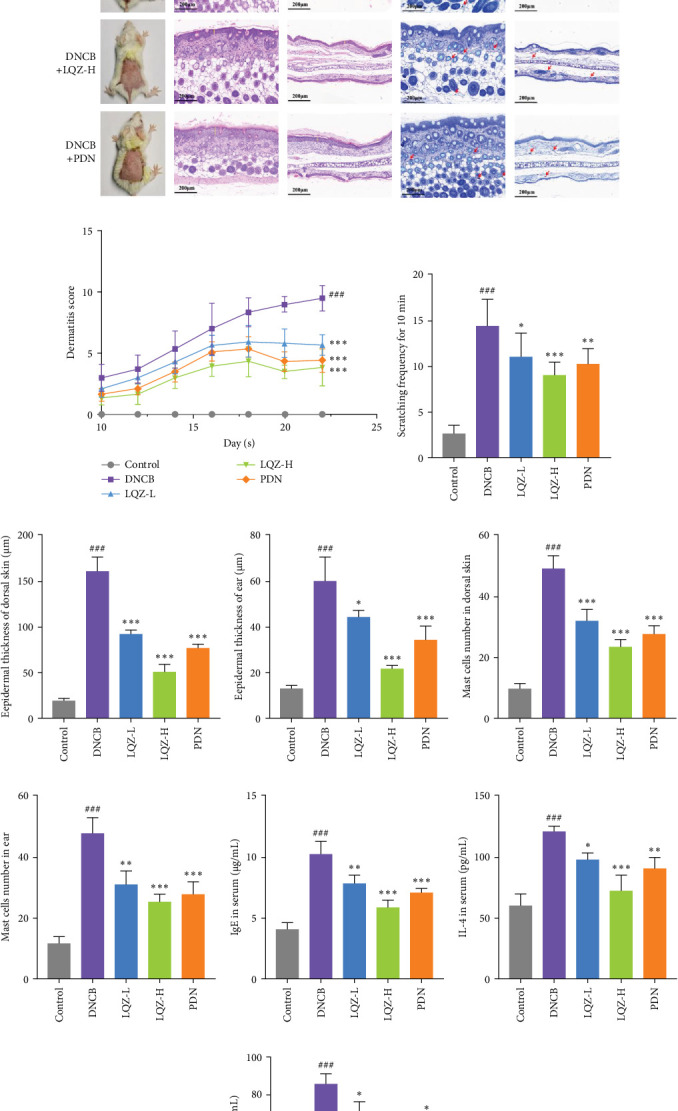
Therapeutic efficacy of LQZ on inflammation in DNCB-induced atopic dermatitis mice. (A) Representative photographs of mouse skin from different groups were displayed. Epidermal thickness, measured using H&E staining under a light microscope (magnification 200×, scale bars 200 μm), was indicated by the yellow straight line, with mast cell infiltration observed through TB staining under the same conditions (magnification 200×, scale bars 200 μm) and indicated by red arrows. (B) Dermatitis scores were performed as described in the methods section. (C) Scratching frequency of mice within 10 min. (D–E) Epidermal thickness of dorsal and ear skin in the different groups. (F–G) Mast cell counts of dorsal and ear skin in the different groups. (H–J) The level of IgE, IL-4, and IL-1*β* in serum was evaluated by ELISA. *N* = 3 mice/group. Data were shown as mean ± SD and analyzed by one-way ANOVA. ^###^*p* < 0.001 versus CON group, *⁣*^*∗*^*p* < 0.05, *⁣*^*∗∗*^*p* < 0.01, *⁣*^*∗∗∗*^*p* < 0.001 versus DNCB group.

By repeatedly applying DNCB to the back and right ear skin of mice, we successfully induced AD-like skin damage. In Figure 2A,B, the back skin lesions of mice in the Control group did not show inflammatory changes. In contrast, the back skin of mice in the DNCB model group exhibited evident signs of inflammation, such as redness, swelling, epidermal peeling, and scab formation, which increased with the number of DNCB applications. In comparison to the model group, mice treated with low and high doses of LQZ, as well as PDN treatment, showed a significant reduction in skin lesions, indicating notable differences. According to the inflammation score results, the recovery of skin lesions was more significant in the LQZ-H treatment group and PDN treatment group. Pruritus is a predominant symptom of AD, and on the 22nd day of the experiment, we recorded the scratching frequency of mice within 10 min to assess the degree of itching. In Figure 2C, the scratching frequency of mice in the model group significantly increased, showing a significant difference compared to the control group. However, after LQZ or PDN treatment, the scratching behavior of mice significantly decreased, indicating that both LQZ and PDN treatments effectively alleviated the itching symptoms in mice.

Abnormal histological changes were observed in the dorsal skin and ears of mice treated with DNCB. In Figure 2A,D,E, H&E staining of the dorsal skin and ear tissues of DNCB-treated mice showed epidermal thickening, increased by 8.3-fold and 4.6-fold, respectively, compared to the control group. In contrast, the LQZ-H group (15.2 g/kg) and LQZ-L group (7.6 g/kg) significantly reduced the thickness of the dorsal epidermis (decreased by 68.4% and 42.7%, respectively) and ear thickness (decreased by 63.8% and 25.8%, respectively), compared to the model group. Meanwhile, PDN demonstrated a reduction of 52.4% in dorsal skin epidermal thickening and 43.1% in ear epidermal thickening.

Furthermore, we stained sections of the dorsal and ear skin with TB to assess the infiltration of mast cells. In Figure 2A,F,G, compared to the control group, the number of mast cells in the dorsal and ear skin increased significantly by 5.1-fold and 4.1-fold, respectively. Compared to the DNCB group, the LQZ-H group (15.2 g/kg) and LQZ-L group (7.6 g/kg) significantly reduced the infiltration of mast cells in the dorsal skin (decreased by 52.4% and 34.7%, respectively) and ear skin (decreased by 46.9% and 35.0%, respectively). In comparison to the DNCB group, the PDN group significantly reduced the infiltration of mast cells in the dorsal and ear skin by 43.5% and 42.0%, respectively. In conclusion, these findings suggest that LQZ treatment effectively alleviates the inflammatory response in the skin lesions of AD mice.

The expression levels of IgE and AD-related inflammatory cytokines IL-4 and IL-1*β* in mouse serum were assessed using the ELISA method. As shown in Figure 2H,I,J, compared to the control group, the levels of IgE, IL-4, and IL-1*β* in mouse serum significantly increased after DNCB induction. However, compared to the DNCB group, the LQZ-H group and LQZ-L group exhibited significant inhibition after treatment. Thus, we conclude that LQZ treatment reduces the expression of IgE and AD-related inflammatory cytokines IL-4 and IL-1*β* in BALB/c AD mice.

### 3.2. General Features of the AD Skin Proteome

To further investigate the potential mechanisms of LQZ in improving AD, we conducted the quantitative proteomic analysis using TMT labeling on mouse dorsal skin tissues from CON, DNCB, PDN, and LQZ-H groups, with three mice per group. We filtered these DEPs using criteria of fold change (FC) ≥1.5 or ≤0.667 (*p* < 0.05).

**Figure 3:**
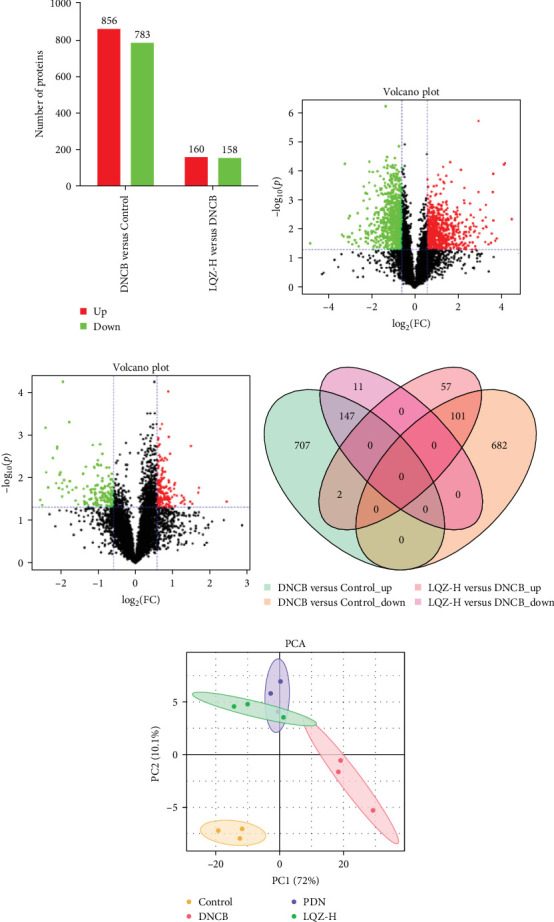
Identification of DEPs in skin tissues of CON, DNCB (model), and LQZ-H groups through TMT-based quantitative proteomic technology. (A) Comparison of the number of DEPs between the DNCB group vs. the CON group and the LQZ-H group vs. the DNCB group. (B) Volcano plot illustrates the DEPs between the DNCB group vs. the CON group. (C) Volcano plot illustrates the DEPs between the LQZ-H group vs. the DNCB group. In the volcano plots, red dots represent upregulated proteins, and green dots represent downregulated proteins, which are filtered based on fold change (FC) ≥1.5 or ≤0.667 and *p* < 0.05. (D) Venn diagram shows the distribution of overlapping DEPs. (E) PCA analysis of the CON, DNCB, PDN, and LQZ-H groups. Each group includes *N* = 3 mice.

In the proteomic analysis, a total of 1639 DEPs were identified between the DNCB group and the CON group, with 685 upregulated and 783 downregulated proteins. In addition, 318 DEPs were discovered between the LQZ-H group and the DNCB group, comprising 158 upregulated and 160 downregulated proteins (Figure 3). Furthermore, 248 overlapping DEPs were identified between the DNCB group versus CON group and the LQZ-H group versus DNCB group (Figure 3D, Supporting Information 1).

Principal component analysis (PCA) of the skin proteome revealed clear separation between the pink triangle control group and the purple cross-shaped model group. Proteins in the LQZ-H and PDN groups were positioned between the model and control groups, suggesting the potential protective effects of LQZ-H and PDN (Figure 3E).

### 3.3. Bioinformatic Analysis of DEPs by Cluster Analysis, PPI Network, GO, and KEGG Enrichment

**Figure 4:**
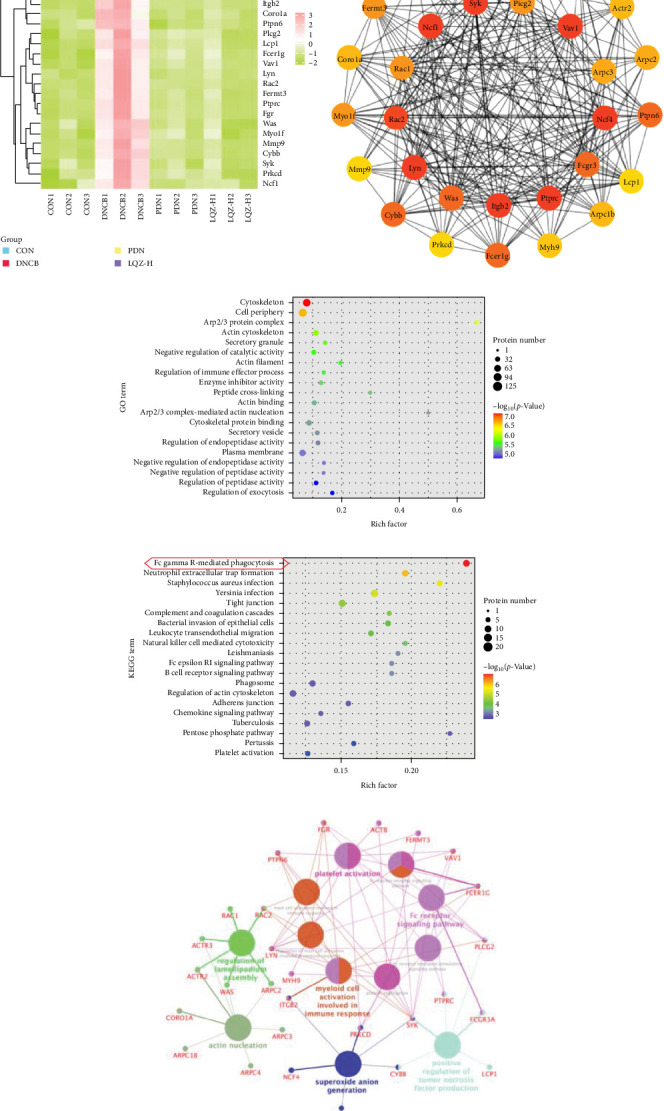
(A) The heatmap illustrates the top 30 DEPs between the LQZ-H versus DNCB. Clusters in pink represent upregulated proteins, while clusters in olive indicate downregulated proteins. The intensity of the color signifies the degree of upregulation and downregulation. The expression of Fcgr3, Lyn, Syk, Plcg2, Ncf1, Rac2, and Arpc3 in the DNCB group were reversed after LQZ-H treatment. (B) PPI network of top 30 DEPs between the LQZ-H versus DNCB. (C) GO enrichment chart showing the top 20 GO terms between the LQZ-H versus DNCB. (D) KEGG enrichment chart showing top 20 pathways between the LQZ-H versus DNCB. (E) The biological processes of the top 30 DEPs were analyzed using Cytoscape's ClueGO and CluePedia plug-ins. In the resulting network, biological pathways are represented by large nodes, while their corresponding target genes or proteins are depicted as smaller nodes, all interconnected. To enhance clarity, the pathway nodes are color-coded. Nodes sharing the same or similar biological functions are grouped together and marked with the same color, allowing for easier visualization of clusters that represent functionally related biological processes.

In our study, the protein–protein interactions (PPI) of 248 intersecting differential proteins were analyzed using the STRING database. This data was then imported into Cytoscape 3.7.1, where we employed the MCC algorithm to assess the centrality of nodes by calculating their maximum clique size. We selected the top 30 target proteins from these 248 for hierarchical clustering, aiming to determine if the groups (DNCB, CON, PDN, and LQZ-H proteins, relative to DNCB, *p* < 0.05) could be differentiated using unsupervised statistical techniques. This approach led to a three-level clustering representing technical replicates, as shown in Figure 4A.

Cluster analysis results suggest that the DEPs, identified by proteomics methods, effectively represent the impact of LQZ-H on AD mice. When the sample groups were clustered based on significantly different proteins, a distinct separation between the DNCB and CON groups was observed, with minimal overlap. The protein levels in the CON, PDN, and LQZ-H groups showed significant similarity compared to the DNCB group. We discovered that a significant portion of the differential proteins are associated with Fc*γ*R-mediated phagocytosis, encompassing 17 key targets: Fcgr3, Syk, Lyn, Plcg2, Ncf1, Vav1, Was, Rac1, Rac2, Actr2, Actr3, Arpc1b, Arpc2, Arpc3, Arpc4, Prkcd, and Ptprc. Following treatment with LQZ-H, there was a notable reversal in the expression levels of these proteins in the DNCB group, with particular emphasis on proteins such as Fcgr3, Lyn, Syk, Plcg2, Ncf1, Rac2, and Arpc3.

Then, we depicted the top 30 target proteins in a network diagram, as shown in Figure 4B. In this diagram, the significance of each protein within the network is indicated by the depth of the node color. Proteins such as Fcgr3, Syk, Plcg2, Lyn, Ncf1, and Rac1/2 are highlighted as crucial components in the process of Fc*γ*R-mediated phagocytosis.

Our GO functional enrichment analysis identified 2931 enriched GO terms, encompassing 2223 biological process (BP) entries, 366 cellular component (CC) entries, and 342 molecular function (MF) entries. The top 20 significant GO terms were illustrated in Figure 4C. The CC analysis revealed that the proteins predominantly located in the cytoskeleton, cell periphery, Arp2/3 protein complex, actin cytoskeleton, and secretory granules. The BP analysis showed that the proteins were mainly involved in processes like negative regulation of catalytic activity, regulation of immune effector processes, peptide cross-linking, actin nucleation mediated by the Arp2/3 complex, regulation of endopeptidase activity, and regulation of exocytosis. The MF classification indicated a significant involvement of these proteins in enzyme inhibitor activity, actin binding, and cytoskeletal protein binding. Our GO analysis results suggest that the DEPs are mainly associated with the cytoskeleton and the Arp2/3 protein complex.

Additionally, we performed an enrichment analysis of the top 20 pathways among 229 identified pathways (Figure 4D). This analysis revealed significant enrichment of the DEPs in various pathways, notably Fc*γ*R-mediated phagocytosis, Neutrophil extracellular trap formation, *S. aureus* infection, complement and coagulation cascades, and bacterial invasion of epithelial cells. Among these, Fc*γ*R-mediated phagocytosis was the most significant in the enriched pathways, closely linked to cytoskeleton reorganization, activation of the Arp2/3 protein complex, and regulation of immune effector processes [17].

Figure 4E presents ClueGO-derived network visualization, where biological pathways (large nodes) and their associated target genes or proteins (small nodes) are interconnected. Pathways are color-coded for clarity, with pink nodes forming a dense cluster that highlights the Fc receptor signaling pathway as a central hub interacting with multiple immune pathways. This underscores its pivotal role in modulating innate and adaptive immune responses, especially through activation of macrophages, neutrophils, and mast cells. Key genes within this pathway, including SYK, PLCG2, FCER1G, LYN, RAC2, and FCGR3A—are essential for immune cell activation, mediating processes like antibody-dependent cell-mediated cytotoxicity (ADCC) and immune cell signaling, which are critical for pathogen defense.

**Figure 5:**
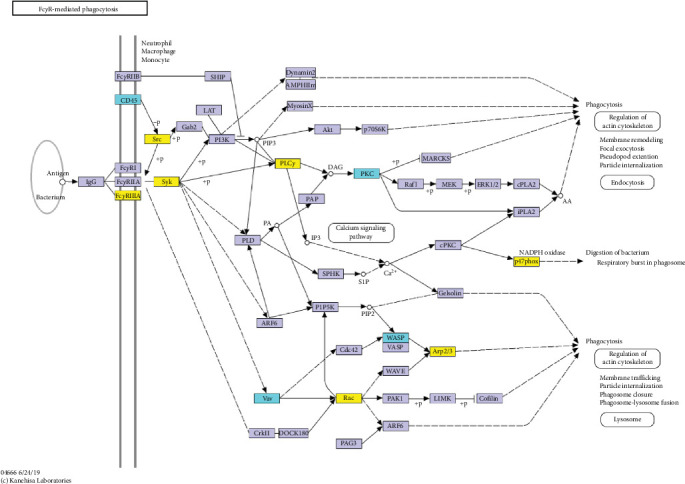
Schematic diagram of LQZ-regulated Fc gamma R-mediated phagocytosis (ko04666) and the involved DEPs (in yellow color).

Based on these findings, we hypothesize that Fc*γ*R-mediated phagocytosis could be a central pathway in the LQZ treatment for AD, with Fcgr3, Tyrosine-protein kinase (Syk), Phosphoinositide phospholipase C-gamma-2 (Plcg2), V-yes-1 Yamaguchi sarcoma viral related oncogene homolog (Lyn), Neutrophil cytosol factor 1 (Ncf1), Ras-related C3 botulinum toxin substrate 2 (Rac2) and Actin-related protein 2/3 complex subunit 3 (Arpc3) emerging as key proteins (Table 2, up-regulated in DNCB group and down-regulated in LQZ-H group). Therefore, we next focused on DEPs of Fcgr3, Lyn, Syk, Plcg2, Ncf1, Rac2, and Arpc3 and the enrichment pathway of Fc*γ*R-mediated phagocytosis (Figure 5).

### 3.4. LQZ Inhibited Fc*γ*R-Mediated Phagocytosis Through Suppressing Fcgr3, Lyn, Syk, Plcg2, Ncf1, Rac2, and Arpc3 Expression in Dorsal Skin Tissues of DNCB-Induced AD Mice

**Figure 6:**
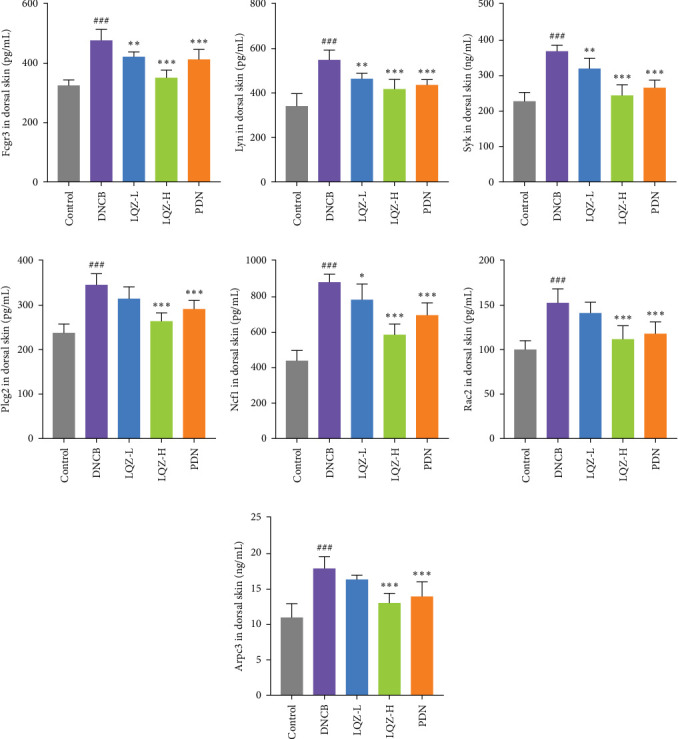
The inhibitory effect of LQZ on proteins related to Fc gamma R-mediated phagocytosis in the skin tissue of AD mice. (A–G) The expression levels of proteins such as Fcgr3, Lyn, Syk, Plcg2, Ncf1, Rac2, and Arpc3 were assessed using ELISA. *N* = 6 mice/group. Data were shown as mean ± SD and analyzed by one-way ANOVA. ^###^*p* < 0.001 versus CON group, *⁣*^*∗*^*p* < 0.05, *⁣*^*∗∗*^*p* < 0.01, *⁣*^*∗∗∗*^*p* < 0.001 versus DNCB group.

The protein levels of Fcgr3, Lyn, Syk, Plcg2, Ncf1, Rac2, and Arpc3 were quantified using ELISA. As depicted in Figure 6A–G, there was a marked increase in these proteins in the dorsal skin tissue of AD mice (*p* < 0.001). Following treatment with a high dose of LQZ, the protein levels of Fcgr3, Lyn, Syk, Plcg2, Ncf1, Rac2, and Arpc3 significantly decreased (*p* < 0.001). With the low-dose LQZ treatment, the protein levels of Fcgr3, Lyn, Syk, and Ncf1 notably decreased (*p* < 0.01 or *p* < 0.05), while the levels of Rac2, Plcg2, and Arpc3 demonstrated a downward trend, though not reaching statistical significance. These findings indicate that high-dose LQZ effectively reduces the expression of proteins involved in Fc*γ*R-mediated phagocytosis in the skin tissue of DNCB-induced mice, contributing to the treatment of AD.

## 4. Discussion

AD stands as one of the most prevalent and thoroughly researched chronic inflammatory skin conditions. The progression of AD is influenced by a combination of factors including impaired skin barrier function, an altered immune system, and a complex genetic background [18]. Patients with AD typically undergo systemic immune activation and inflammatory responses. While topical or systemic corticosteroids are commonly used in AD treatment, their prolonged use can result in various adverse reactions such as skin atrophy, telangiectasia, purpura, striae, and hyperpigmentation [19]. Emerging therapies like dupilumab or JAK inhibitors offer new treatment avenues for moderate to severe AD, yet their long-term efficacy in management is still under investigation [20, 21]. Recent studies highlight the potential of TCM as an alternative or complementary approach in managing AD, with the possibility of enhancing patient quality of life [22–24]. LQZ is a TCM prescription used for treating AD. While previous studies have primarily focused on LQZ's clinical efficacy, cytokine modulation, and key ingredients [11, 15]. this study is the first to employ TMT-based quantitative proteomics to systematically map global protein changes in AD skin tissue. Through this approach, we identified Fc*γ*R-mediated phagocytosis as a novel pathway involved in LQZ's therapeutic effects, an aspect previously unrecognized in its mechanism of action. For the first time, we demonstrate that LQZ suppresses critical proteins in the Fc*γ*R-mediated phagocytosis (Fcgr3, Lyn, Syk, Plcg2, Ncf1, Rac2, and Arpc3), which are essential for recognition and binding of immune complexes, intracellular signal transduction, activation of phagocytic cells, cytoskeletal reorganization, and the phagocytosis process itself [25–28]. Notably, this pathway has never been linked to LQZ or any other TCM formula for AD.

In our study, we observed notable anti-inflammatory and skin barrier protective effects of LQZ in DNCB-induced AD mice. AD is typically characterized by epidermal thickening, cellular edema, and infiltration of lymphocytes, mast cells, and eosinophils in the dermis [29]. H&E and TB staining revealed thickened epidermis and mast cell infiltration in the back and ears of the AD group, which were effectively reversed by LQZ in a dose-dependent manner. In AD patients, damage to the epidermal barrier results in an imbalance of immune-regulatory proteins and the release of damage-associated molecular patterns, including alarmins like IL-1*β* and IL-33 [3]. These mediators further stimulate and activate group 2 innate lymphoid cells (ILC2) and Th2, leading to immune responses in the skin. Activated Th2 cells release IL-4 and IL-13, fostering the transition of B cells to IgE class antibodies and the production of specific IgE [18]. Our study found that LQZ treatment significantly reduced the levels of IgE, IL-4, and IL-1*β* in the serum of AD mice. These findings suggest that LQZ possesses anti-inflammatory and skin barrier protective pharmacological properties, showing potential as a therapeutic agent for AD.

To delve deeper into the potential mechanisms underlying the efficacy of LQZ in treating AD, we carried out a TMT-based quantitative proteomics study. Through this study, we identified 1639 high-quality, quantifiable proteins. Out of these, 248 were found to be DEPs in the LQZ-H group compared to the AD group. These DEPs formed the basis for our subsequent bioinformatics analysis. KEGG pathway enrichment analysis showed a significant enrichment of differential DEPs in the Fc*γ*R-mediated phagocytosis pathway, indicating its critical role in LQZ's therapeutic effects on AD. Further analysis identified key proteins associated with the Fc*γ*R-mediated phagocytosis signaling pathway, including Fcgr3, Syk, Plcg2, Lyn, Ncf1, Rac2, and Arpc3. Fc*γ*R-mediated phagocytosis is essential for pathogen elimination, autoantigen clearance, immune response regulation, and ADCC [30]. These functions are pivotal in maintaining immune homeostasis and providing defense against infections.

UPLC-MS/MS analyses [13] identified 49 serum-absorbed metabolites in LQZ, including L-arginine, L-phenylalanine, matrine, sophocarpine, *α*-hydroxylinoleic acid, and caffeic acid. These bioactive components are known to exhibit anti-inflammatory properties and enhance skin barrier function, thereby alleviating AD [31–36]. The therapeutic efficacy of LQZ in AD is likely attributable to the synergistic effects of these active ingredients and the combined application of herbal components. While prior studies have characterized the active constituents of LQZ, their direct contribution to the Fc*γ*R-mediated phagocytic mechanism identified in this study remains undetermined. Future investigations, such as metabolomic profiling and targeted compound validation, are necessary to establish direct links between these bioactive compounds and the observed therapeutic outcomes [37, 38].

Fc*γ*R receptors (Fc*γ*Rs), surface proteins expressed on immune cells like macrophages, neutrophils, dendritic cells, and B cells, are pivotal regulators of humoral and innate immunity [17]. These receptors are classified into subtypes (e.g., Fc*γ*RI, Fc*γ*RII, and Fc*γ*RIII), each exhibiting distinct functions and cell-type specificity [39]. In AD, Kiekens reported an increased expression of Fc*γ*RI (CD64) and Fc*γ*RIII (CD16) in acute and chronic dermatitis lesions compared to healthy and non-lesional AD skin [40]. While IgE and its receptor (Fc*ε*RI) are central to allergic reactions, IgG mainly interacts with Fc*γ*RIIIA and Fc*γ*RIV. It was demonstrated that mast cells significantly contribute to Fc*γ*RIIA-dependent passive cutaneous anaphylaxis in mice. Additionally, monocytes, macrophages, and neutrophils were crucial in Fc*γ*RIIA-dependent passive systemic anaphylaxis [41].

Fc*γ*R-mediated phagocytosis in macrophages is regarded as a crucial process in initiating inflammation [42]. In AD, macrophages are observed in acute and chronic inflammatory skin conditions, where they play an integral role in immune responses [43]. These macrophages substantially affect AD's progression by producing inflammatory cytokines and growth factors and acting as antigen-presenting cells [44]. In AD patients, macrophage function may become dysregulated, leading to an increased susceptibility to microbial infections [45]. Fc*γ*Rs on macrophages and dendritic cells recognize immune complexes, facilitating pathogen clearance and modulating skin inflammation [46]. Upregulation Fc*γ*Rs expression might lead to a stronger inflammatory response[40]. M2 macrophages are typically activated by Th2 cell cytokines IL-4/IL-13 and are characterized by their role in suppressing inflammatory factors, thus, inhibiting inflammatory responses and facilitating tissue remodeling [47]. Type 2 inflammation, commonly associated with AD, allergic reactions, asthma, and certain autoimmune diseases, has been increasingly linked to Fc*γ*Rs and Th2 immune responses [48]. A study demonstrated that macrophages, upon simultaneous stimulation by activated Fc*γ*Rs and Toll-liAke receptors (TLRs), entered a distinct M2b or regulatory activation state, exhibiting a unique cytokine profile with reduced IL-12 and increased IL-10, tumor necrosis factor (TNF), IL-1, and IL-6, indicating a shift towards a more pro-inflammatory state [49]. These findings imply that targeting Fc*γ*R-mediated phagocytosis inhibition could be a promising strategy for treating AD.

The IgG Fc region receptors (Fc*γ*Rs) on macrophages play a pivotal role in host immunity, primarily involved in antibody-dependent cellular phagocytosis and immune regulation [50]. CD16 (Fcgr3), essential for antigen presentation and immune activation [51], is implicated in AD pathogenesis: CD16-deficient animal models exhibit attenuated skin inflammation and humoral responses [52], alongside suppressed Th2/Th1/Th17 immunity and reduced inflammatory cytokines [52]. Fc*γ*R signaling is initiated by Src-family kinase Lyn, which phosphorylates immunoreceptor tyrosine-based activation motifs (ITAMs) upon ligand binding [26, 53 ], triggering Syk, protein kinase C, and calcium-dependent cascades that amplify immune signals [54].

It was reported that Lyn and Syk proteins, along with their phosphorylated forms, are associated with AD [55, 56], and their inhibition has shown anti-inflammatory and anti-allergic effects, potentially improving AD symptoms [57–59]. Reducing the phosphorylation levels of Lyn and Syk was confirmed to effectively inhibit the increase in intracellular Ca^2+^ concentration, cytokine production, and histamine release [55, 60], leading to controlled mast cell degranulation and contributing to the improvement of AD. Studies also showed that Lyn played a crucial role in regulating Th2 responses, as Lyn-deficient mice exhibit resistance to the effects of reduced Th2 immune function [61 ]. Additionally, inhibiting Syk was found to enhance the effects of JAK inhibitors, providing further therapeutic benefits in the treatment of AD [62, 63]. When activated and phosphorylated by Syk, Plcg2 (PLC*γ*2) initiates the catalysis of diacylglycerol and inositol trisphosphate. This process results in the release of Ca^2+^ from the endoplasmic reticulum [64]. The released secondary messengers further stimulate various intracellular signaling pathways, thereby enhancing the activation and phagocytic capabilities of phagocytic cells. In macrophages, the increase in Ca^2+^ induced by Fc*γ*R activation relies on Plcg2. Intriguingly, inhibiting this Ca^2+^ surge modifies the Fc*γ*R-mediated inflammatory response without impacting its phagocytic function [65 ]. Mice lacking Plcg2 demonstrated resistance to IgE-mediated skin inflammatory responses, highlighting the crucial role of Plcg2 in Fc*ε*R-mediated skin inflammation [66]. Furthermore, research has also established the essential role of PLC*γ*2 in Fc*γ*R-mediated passive skin inflammatory responses [66].

Rac2 and Arpc3 function synergistically to promote cytoskeletal reorganization and cellular morphological alterations, thereby enhancing Fc*γ* receptor-mediated phagocytosis [27, 28, 67]. Studies established the effectiveness of narrowband ultraviolet B (NB-UVB) therapy in managing skin conditions, notably AD [20]. Furthermore, within 12 h of NB–UVB irradiation, the expression of cytoskeletal-related proteins, including FN1, ITGB4, ITGA1, RAC2, and DOCK1, significantly decreased [68]. Neutrophils are pivotal in pathogen defense, employing mechanisms such as the release of extracellular DNA traps, pathogen phagocytosis, and reactive oxygen species (ROS) production. Ncf1 (neutrophil cytosolic factor 1, also known as p47phox), a component of the NADPH oxidase complex, plays a vital role in generating ROS. These species are essential for eradicating ingested pathogens and modulating inflammatory responses [69]. A deficiency in Ncf1 leads to diminished oxidative bursts during Fc*γ*R-mediated phagocytosis, consequently impairing the bactericidal effectiveness of phagocytes [69]. Prior research established a close connection between AD and elevated levels of oxidative damage, coupled with diminished antioxidant defense capabilities [70]. Controlling the production of ROS could diminish the activation of inflammatory cells and the release of inflammatory mediators, which in turn aids in alleviating the inflammatory symptoms observed in patients with AD.

The aforementioned studies highlight the pivotal roles of Fcgr3, Lyn, Syk, Plcg2, Ncf1, Rac2, and Arpc3 in Fc*γ*R-mediated phagocytosis, underlining their importance in the pathogenesis of AD. Consequently, we utilized ELISA to measure the expression levels of these proteins. The results indicated that in the skin tissue of AD group mice, there was a significant upregulation in the expression of these proteins. However, following LQZ-H treatment, their expression levels notably decreased. Therefore, LQZ-H effectively inhibits the expression of Fcgr3, Syk, Plcg2, Lyn, Ncf1, Rac2, and Arpc3, thereby reducing Fc*γ*R-mediated phagocytosis.

While this study offers novel insights into the anti-AD mechanisms of LQZ, several limitations warrant acknowledgment. First, although the DNCB-induced AD mouse model recapitulates key pathological features of human AD, such as Th2-driven inflammation and skin barrier dysfunction, it fails to fully represent the diversity of human immune responses or the complexities of long-term disease progression. Second, the long-term efficacy and safety of LQZ require further investigation, particularly regarding its ability to provide sustained symptom relief and prevent relapse. Prospective, multi-center clinical trials are essential to evaluate its long-term effectiveness in diverse patient populations with AD. These studies should incorporate longitudinal biomarker analysis (Fcgr3, Lyn, Syk, Plcg2, Ncf1, Rac2, and Arpc3) to identify patient subgroups most likely to benefit from LQZ and assess the durability of its therapeutic effects. Third, preclinical chronic toxicity studies should evaluate potential organ toxicity, such as hepatic and renal dysfunction, while long-term clinical surveillance is critical for detecting rare adverse effects. Addressing these gaps in future research will enhance LQZ's translational potential and its role in personalized AD management.

## 5. Conclusion

In conclusion, our findings indicate that LQZ notably ameliorates skin barrier damage and inflammatory responses in DNCB-induced AD mice. The therapeutic efficacy of LQZ appears to be associated with its ability to inhibit the expression of proteins such as Fcgr3, Lyn, Syk, Plcg2, Ncf1, Rac2, and Arpc3, which are involved in Fc*γ*R-mediated phagocytosis. This suggests that these proteins have the potential to be effective targets in AD treatment. Our study confirms the anti-inflammatory properties and skin barrier protective effects of LQZ in AD, laying a foundation for its clinical application.

## Figures and Tables

**Table 1:** Linear gradient elution conditions.

*T* (min)	A	B	V
	(0.1% FA, H2O)	(0.08% FA, 80%ACN, H2O)	(nL/min)
0	94%	6%	600
2	91%	9%	600
10	87%	13%	600
50	74%	26%	600
70	62%	38%	600
71	0%	100%	600
78	0%	100%	600

**Table 2:** DEPs associated with Fc gamma R-mediated phagocytosis and regulated by LQZ.

Protein accession	Gene name	DNCB/CON *p*-Value	DNCB/CON log2 (FC)	DNCB/CON Regulation type	LQZ-H/ DNCB *p*-Value	LQZ-H/ DNCB log2 (FC)	LQZ-H/ DNCB type
P08508	Fcgr3	0.009174147	2.878674506	Up	0.015068904	0.494430004	Down
E9PWE9	Syk	0.026901954	1.893842887	Up	0.029974095	0.540919283	Down
P25911	Lyn	0.022156753	2.304435484	Up	0.032721054	0.486789151	Down
Q8CIH5	Plcg2	0.01786896	2.347063979	Up	0.024139943	0.472927558	Down
Q09014	Ncf1	0.016814139	2.524781341	Up	0.015681652	0.479022325	Down
Q05144	Rac2	0.021955592	4.747113799	Up	0.028187086	0.256514186	Down
Q9JM76	Arpc3	0.00196805	2.086092715	Up	0.016191807	0.568707483	Down

## Data Availability

The data that support the findings of this study are available from the corresponding author upon reasonable request.

## Nomenclature

AD: Atopic Dermatitis
ADCC: antibody-dependent cell-mediated cytotoxicity
Arpc3: Actin-related protein 2/3 complex subunit 3
CON: Control
DEPs: differentially expressed proteins
DNCB: 2,4-dinitrochlorobenzene
Fcgr3: Fc-gamma RIII
H&E: hematoxylin and eosin
LQZ: Liangxue Qushi Zhiyang Decoction
LQZ-H: high-dose LQZ treatment
LQZ-L: low-dose LQZ treatment
Lyn: V-yes-1 Yamaguchi sarcoma viral related oncogene homolog
Ncf1: Neutrophil cytosol factor 1
PCA: Principal component analysis
PDN: Prednisolone group
Plcg2: Phosphoinositide phospholipase C-gamma-2
Rac2: Ras-related C3 botulinum toxin substrate 2
ROS: reactive oxygen species
Syk: Tyrosine-protein kinase
TB: toluidine blue
TCM: traditional Chinese medicine
TMT: labeling and nanoLC-MS/MS tandem mass tag labeling and nano-liquid chromatography-tandem mass spectrometry

## Supporting Information

Supporting Information Supporting Information Table S1: Proteins upregulated in the DNCB group compared to the CON group and downregulated after treatment with LQZ-H. Table S2: Proteins downregulated in the DNCB group compared to the CON group and upregulated after treatment with LQZ-H.
